# Up-regulation of *LINC00467* promotes the tumourigenesis in colorectal cancer

**DOI:** 10.7150/jca.32216

**Published:** 2019-10-19

**Authors:** Xiaoyun He, Shen Li, Bingbing Yu, Gaoyan Kuang, Yongrong Wu, Meili Zhang, Yuxiang He, Chunlin Ou, Pengfei Cao

**Affiliations:** 1 Department of Pathology, Xiangya Hospital, Central South University, Changsha, Hunan 410008, China.; 2 Department of Endocrinology, Xiangya Hospital, Central South University, Changsha, Hunan 410008, China.; 3 Department of Pathology, Dezhou People's Hospital, Dezhou, Shandong 253056, China; 4 Department of Orthopedics, The First Affiliated Hospital of Hunan University of Chinese Medicine, Changsha, Hunan 410007, China.; 5 Department of Oncology, Xiangya Hospital, Central South University,Changsha, Hunan 410008, China.; 6 Department of Hematology, Xiangya hospital, Central South University, Changsha, Hunan 410008, China.; 7 National Clinical Research Center for Geriatric Disorders, Xiangya Hospital, Central South University, Changsha 410008, Hunan, China.

**Keywords:** *LINC00467*, colorectal cancer, tumorigenesis, invasion, survival

## Abstract

Recent studies have reported that long non-coding RNAs (lncRNAs) are associated with the tumourigenesis of colorectal cancer (CRC); however, several of these are yet to be identified and characterised. In this study, we report a novel lncRNA, *LINC00467*, which was significantly up-regulated in CRC; we investigated its function and mechanism in CRC. Our study demonstrated that LINC00467 levels in 45 pairs of CRC tissues were higher than those in the corresponding normal colon mucosal tissues. We used the Gene Expression Omnibus (GEO) and Gene Expression Profiling Interactive Analysis (GEPIA) databases for the analysis and measurement of clinical samples, and observed that the CRC patients with high *LINC00467* expression levels showed poor overall survival (OS) and recurrent-free survival (RFS) rates. Furthermore, following the short interfering RNA (siRNA) knockdown of *LINC00467* in the CRC cell line, the results demonstrated that *LINC00467* suppresses the proliferation, invasion and metastasis of CRC cells *in vitro*. Moreover, its molecular mechanism of *LINC00467* decreased the expression of Cyclin D1, Cyclin A1, CDK2, CDK4 and Twist1 as well as enhanced the expression of E‑cadherin. Collectively, these findings suggest that *LINC00467* may be crucial in the progression and development of CRC, and may serve as a potential therapeutic target for CRC patients.

## Introduction

Colorectal cancer (CRC) is the second leading cause of cancer-related mortalities in the United States and the third most commonly diagnosed malignancy worldwide 1-4. In the past decades, improvements in the therapeutic approach to CRC, have led to substantial progress in treating CRC. However, CRC-associated mortality has not changed as expected 5, 6. The progression and development of CRC may involve a multi-step process, majorly including the inherited and environmental factors that eventually result in a series of gene mutagenesis events associated with cancer cell apoptosis, proliferation, and differentiation 7, 8. With the popularity of gene tests and molecular therapy, several studies have investigated the molecular pathogenesis of CRC by analysing the molecular abnormalities in CRC progression and development 9-11.

Long non-coding RNAs (lncRNAs) form a subset of RNAs. Their transcribing length is more than 200 nucleotides (nt), and they lack a functional open reading frame (ORF); however, rarely, they may encode a functional short peptide 12, 13. Recently, lncRNAs have been widely studied due to the role they play in various human diseases, including cancer. Several studies revealed that lncRNAs were aberrantly expressed in various cancers, such as tongue squamous cell carcinoma (TSCC) 14, CRC 15, gastric cancer 16, cervical cancer 17 and hepatocellular cancer 18. These abnormally expressed lncRNAs acted as biomarkers for cancer screening, analysis and therapy.

In this study, we analysed two previously published online datasets to find the dysregulated lncRNAs in CRC. One novel lncRNA, long inter-genic non-coding RNA 467* (LINC00467)* was significantly overexpressed in the two CRC datasets. *LINC00467* is located in the chr1q32.3 region, and measures 2616 nt in length; however, little is known about the function and mechanism of *LINC00467* in tumourigenesis. In this study, we determined that the *LINC00467* was overexpressed in CRC tissues and cell lines, and via the Gene Expression Profiling Interactive Analysis (GEPIA) database, we observed that CRC patients with high *LINC00467* expression levels demonstrated poor overall survival (OS) and recurrent-free survival (RFS). Furthermore, *LINC00467* knockdown inhibits the proliferation and metastasis of colorectal cancer cells in vitro. Collectively, these results suggest that *LINC00467*, as a potential prognostic biomarker, is crucial in CRC progression and development.

## Materials and methods

### Tissue samples and Bioinformatic analyses

In total, 45 pairs of CRC tissues and corresponding normal colorectal tissues were collected from newly diagnosed CRC patients at the Xiangya Hospital of Central South University in Changsha, Hunan of China. None of these patients received routine radiotherapy. This study was approved by the Research Ethics Board of Xiangya Hospital of Central South University, and signed informed consent was obtained from each participant before they were enrolled in the study.

All microarray expression data containing primary colorectal cancer data and their correlated clinical data, were deposited in the Gene Expression Omnibus (GEO) database: GSE22598 19, GSE37364 20, and GSE50760 21 (Affymetrix Human Genome U133 Plus 2.0 platform). GSE22598 contained 17 pairs of CRC and adjacent non‑tumour tissues; GSE37364 had 38 normal colon samples and 56 primary CRC samples; GSE50760 had 18 metastasis CRC samples and adjacent non-metastasis samples.

### Cell culture and transfection

The human normal colon mucosal cell line NCM460 and human colorectal cancer cell lines(HT29, SW480, SW620, HCT116) used in this study were obtained from the American Type Culture Collection (ATCC, Manassas, USA). HCT116 cells were maintained in DMEM (Dulbecco's Modified Eagle's medium) with 10% fetal bovine serum (FBS, Invitrogen, USA), and other cell lines (NCM460, SW480, SW620, HT29) were cultured in RPMI-1640 media (Invitrogen, USA) supplemented with 10% FBS. When cell densities were about 60%, 50nM siRNA oligos were transfected by Lipofectamine 3000 (Invitrogen, USA), according to the manufacturer's instructions. The sequences of the *LINC00467* targeting siRNAs were: *LINC00467*-si-1: 5'- GAAGAAGAGAAGAGAGAAA-3'; *LINC00467*-si-2: 5' - -GATAAGAAGTCCACTCACA-3'; sequences of non-target scramble controls were provided by RiboBio(Guangzhou, China).

### Quantitative real-time PCR

RNA isolation and amplification were performed as described previously 22. Thereafter, RNA was reverse transcribed to cDNA via a Revert Aid First Strand cDNA Synthesis kit (Fermentas, Canada). Quantitative real-time PCR (qRT-PCR) was performed using a SYBR_Premix ExTaq II kit (Takara, China) and CFX96 Real-Time PCR Detection System (Bio-Rad, USA) in order to determine the relative expression levels of the target genes. The qRT-PCR primer sequences are shown in Table 1.

### Subcellular Fractionation Analysis

To determine the cellular localization of *LINC00467*, cytoplasmic and nuclear RNA were separated and collected via the PARIS Kit (Invitrogen, USA), according to the manufacturer's instructions. The computing method was used as described previously 23.

### Western blotting

Lysis, electrophoresis, and target protein visualisation were performed as described previously 22. Briefly, the cell lysates (50 μg) were separated by 10% sodium dodecyl sulphate-polyacrylamide gel electrophoresis (SDS-PAGE) and were then transferred onto a polyvinylidene fluoride (PVDF) membrane. Thereafter, the membranes were blocked in 5% defatted milk for 1 h, and incubated overnight at 4 °C with primary anti-cyclinD1, CDK4, Twist1, E‑cadherin and GAPDH antibodies (Cell Signalling Technology, Danvers, MA, USA). The next day, the membranes were incubated with horseradish peroxidase-conjugated secondary antibodies (Santa Cruz Biotechnology) for 1 h at room temperature. The signal was visualised via the ECL detection system, and densitometric analysis of the immunodetected bands was carried out using Image J software (http://rsb.info. nih.gov/ij).

### Cell proliferation assay

Cell proliferation assay was carried out using CCK-8 (Dojin Laboratories, Japan), as described previously 24. Each experiment was repeated thrice independently.

### Flow cytometry for cell cycle analysis

After transfecting with si-NC or si-LINC00467 for 48 h, approximately 1 × 10^6^ HT29 cells were collected for cell cycle distribution, and the DNA content was detected using propidium iodide (PI) (Sigma, San Antonio, USA) staining, according to the methods described in a previous study 25. Cell cycle distribution was analysed via flow cytometry (Beckman Coulter, South Kraemer, USA) using cell Modifit software. Each experiment was repeated thrice independently.

### Transwell Matrigel assays

The invasiveness of the CRC cells was determined by an assay performed in a 24 well Transwell plate (8 μM pore size; Costar), as described previously 26. Briefly, 5 × 10^4^ cells were placed on the upper chamber of each insert coated with 200 mg/ml of Matrigel (BD Biosciences, CA, USA). After 48 h, the invaded cells were stained with haematoxylin and enumerated. Each experiment was repeated thrice independently.

### Statistical analysis

All statistical analyses were carried out using the SPSS version 18.0 software and GraphPad prism version 6.0. Data are presented as mean ± standard error of mean. Differences between the two independent groups were tested with the Student's t-test. OS and RFS were calculated using the Kaplan-Meier method, and the results of a log-rank test were considered significant if *p* < 0.05.

## Results

### LINC00467 is highly expressed in the CRC tissues and cell lines

**Figure 1:**
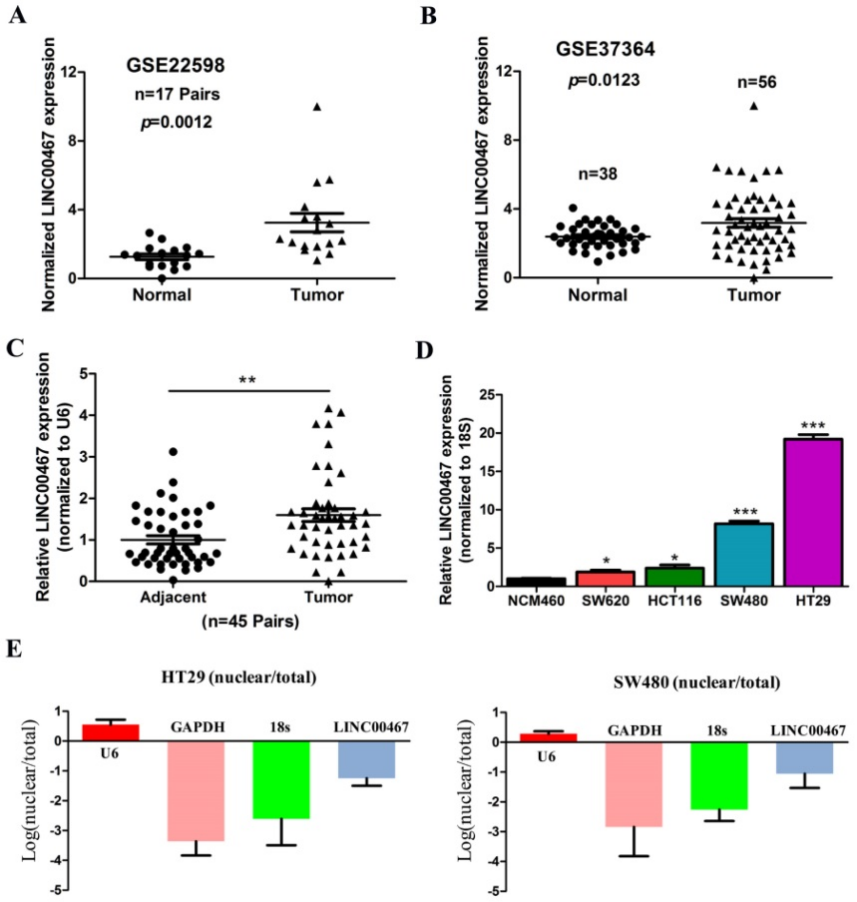
*LINC00467* expression is up-regulated in CRC tissues and cell lines. (A) #GSE22598 (containing 17 pairs of CRC tissues and corresponding normal colorectal tissues) and (B) #GSE37364 (38 normal colon samples and 56 primary CRC samples) from the GEO database were used to analyse the expression of *LINC00467*. (C) *LINC00467* is highly expressed in 45 pairs of CRC tissues compared with the corresponding normal colorectal tissues. (D) *LINC00467* expression was significantly increased in the CRC cell lines (SW480, SW620, HT29 and HCT116) compared with NCM460, a normal colon mucosal cell line. (E and F) Nucleic and cytoplasmic RNA were analysed using qRT-PCR to detect the expression levels of *LINC00467* in HT29 and SW480 cells. U6 was used as a nucleic RNA control; GAPDH and β-actin were used as cytoplasmic RNA controls. Data are presented as mean ± SEM. *p < 0.05 compared with control.

To find the dysregulated lncRNAs in CRC, two online GEO datasets (#GSE22598 and GSE37364) based on the Affymetrix Human Genome U133 Plus 2.0 platform were analysed. One novel lncRNA *LINC00467* was significantly up-regulated in the CRC tissues compared with the non-tumour tissues according to the GSE22598 and GSE37364 datasets (*p* < 0.05, Fig. 1A and B). Furthermore, we detected *LINC00467* expression in 45 pairs of CRC and adjacent non-tumour tissues, and found that the *LINC00467* expression was higher in CRC tissues than in adjacent non-tumour tissues (*p* < 0.05, Fig. 1C). Next, *LINC00467* expression levels were also determined by qRT-PCR in four CRC cell lines (SW480, HT29, HCT116 and SW620) and the normal colon mucosal cell line NCM460. The results revealed that *LINC00467* expression was higher inCRC cell lines than in NCM460 (*p* < 0.05, Fig. 1D), and that it was the highest in HT29 cells. Moreover, we analysed the distribution proportion of *LINC00467* expression in the nucleus and cytoplasm, and found that *LINC00467* expression was higher in the cytoplasm than the nucleus, indicating that subcellular localization of *LINC00467* in CRC cells was majorly located in the cytoplasm (Fig. 1E).

### LINC00467 up-regulation is associated with poor prognosis in CRC

**Figure 2:**
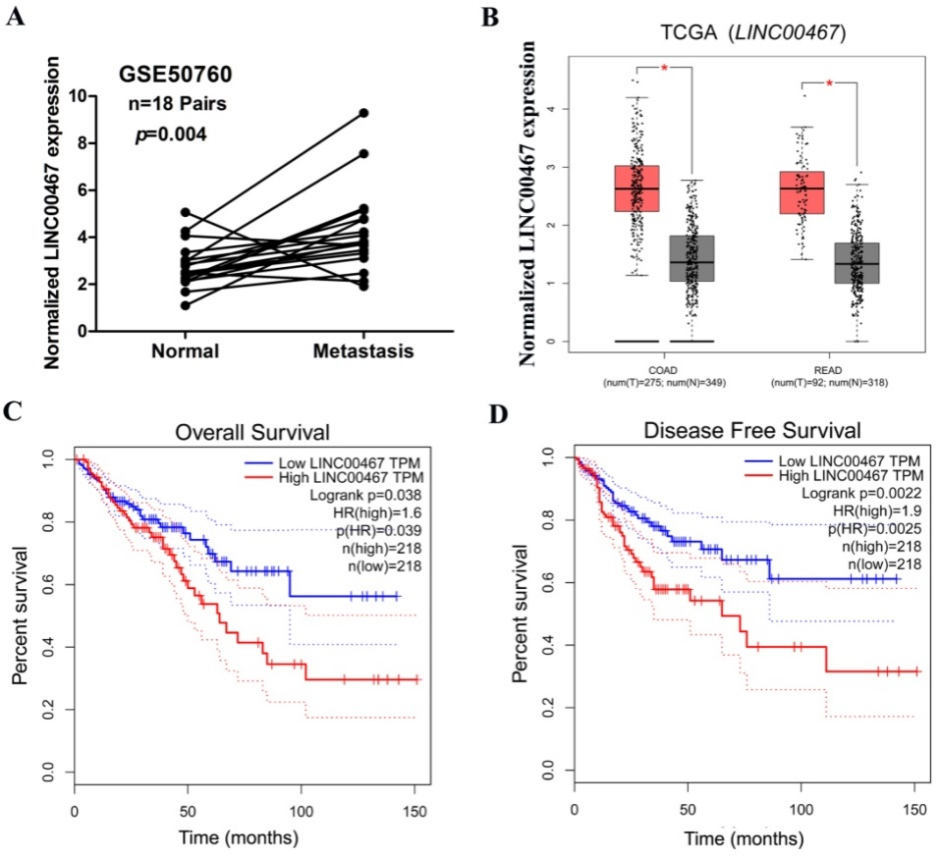
Relationship between *LINC00467* expression and clinicopathological features. (A) Relative expression of *LINC00467* in normal and metastatic human CRC tissues from the GEO database (#GSE50760, 18 metastasis CRC samples and adjacent non-metastatic samples) was studied. (B) The GEPIA database was used to analyse *LINC00467* expression in the CRC samples (COAD stands for colon adenocarcinoma and READ for rectal adenocarcinoma). (C-D) GEPIA database was used to analyse the clinical impact of* LINC00467* expression patterns on the CRC patients' RFS and OS (the TCGA CRC specimens were divided into two groups within the GEPIA database, group 1 = low LINC00467 expression, n = 218; group 2 = high LINC00467 expression, n = 218). *p < 0.05 compared with control.

We next assessed the correlation of *LINC00467* expression with the clinicopathological features of CRC, wherein we assessed the correlation between *LINC00467* expression and distant metastasis in CRC tissues, which was previously analysed by one published dataset (#GSE50760) using Affymetrix HG_U133 Plus 2 arrays. The analysis indicated that higher expression of *LINC00467* significantly correlated with distant metastasis in CRC (*p* = 0.004, Fig. 2A). Thereafter, by accessing the Cancer RNA-Seq Nexus (CRN) database 27, we found that *LINC00467* expression in the pathological stages (I-IV) of CRC was higher than that observed in normal tissues (Table 2). By analysing the public CRC datasets in the GEPIA database 28, we found that *LINC00467* was significantly up-regulated in the colon and rectal adenocarcinoma samples (all *p* < 0.05, Fig. 2B), and that high* LINC00467* expression was associated with poor RFS and OS (all *p* < 0.05, Fig. 2C and D). Collectively, these data indicate that high *LINC00467* expression levels are an independent risk factor for CRC patients.

### Knockdown of LINC00467 expression inhibits cell proliferation and invasion in CRC

**Figure 3:**
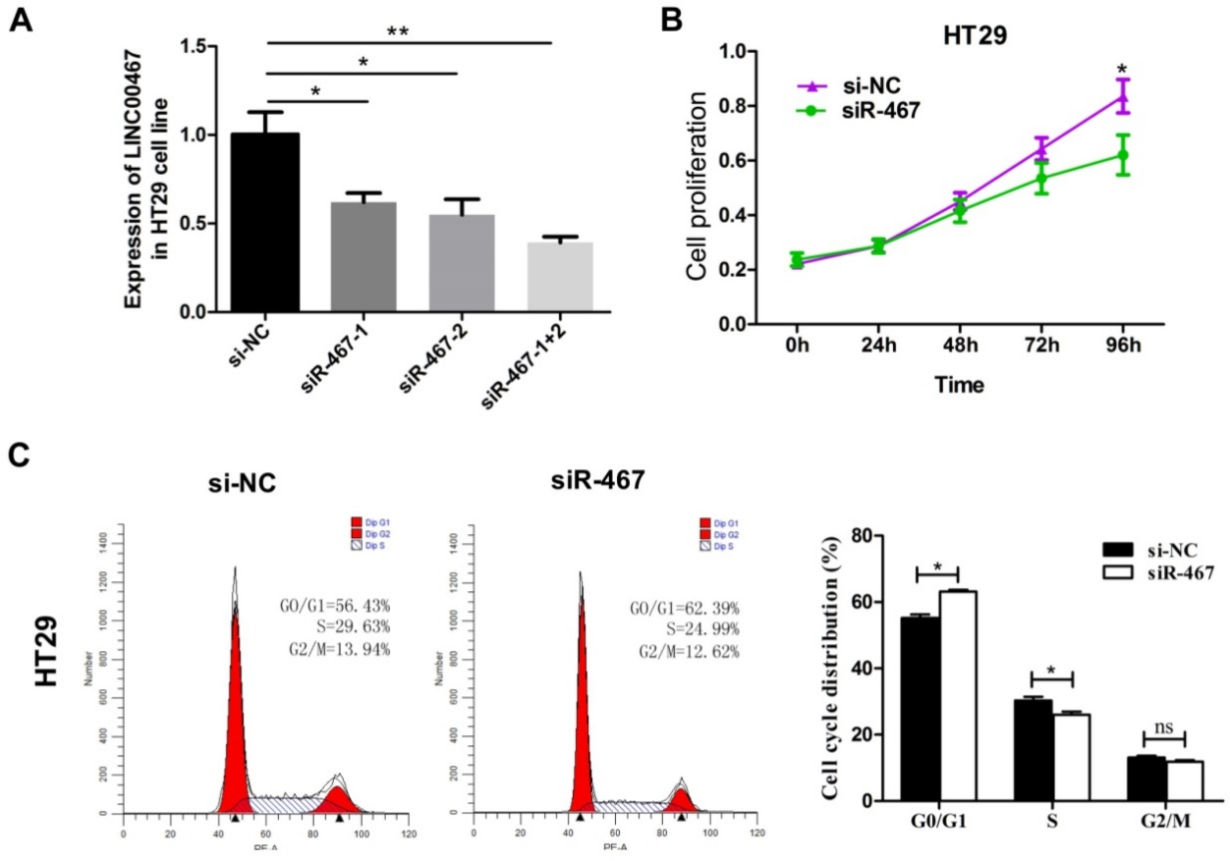
Knockdown of *LINC00467* expression impeded the proliferation of CRC cells*.* (A) The interference efficiency of *siR-467* was verified in HT29 cells. HT29 cells were transfected with either *si-NC* or *siR-467* (1#, 2#, 1+2#) for 48 h, and then, *LINC00467* expression was analysed by qRT-PCR. After transfecting HT29 cells with *si-NC* or *siR-467* for 48 h, the CCK8 assay (B) and flow cytometry (C) were used to detect the cell proliferative ability. Data are presented as mean ± SEM. *p < 0.05, **p < 0.01 compared with control.

To verify *LINC00467* function in CRC cells, we first measured the efficiency of the short interfering RNA (siRNA), *siRNA-LINC00467*(*siR-467*). The results demonstrated that the siR-467-1+2 group, when compared with both siR-467-1 and siR-467-2 groups revealed the highest interfering efficiency in HT29 cells (Fig. 3A). Therefore, we transfected the siRNA-LINC00467-1+2 in HT29 cells to detect and analyse the change in biological function. After investigating the siRNA efficacy, we assessed the biological function induced by* LINC00467* knockdown in the CRC cells. Initially, we explored the effect of *LINC00467* knockdown on the proliferation of CRC cells. By performing CCK-8 proliferation assays, we found that knocking down *LINC00467* expression significantly inhibited HT29 cell proliferation relative to that of the control cells (*p* < 0.05, Fig. 3B). Meanwhile, flow cytometry analysis revealed that knocking down *LINC00467* expression in HT29 cells elevated the percentage of cells in the G1 phase and reduced the percentage of cells in the S phase (*p* < 0.05, Fig. 3C).

**Figure 4:**
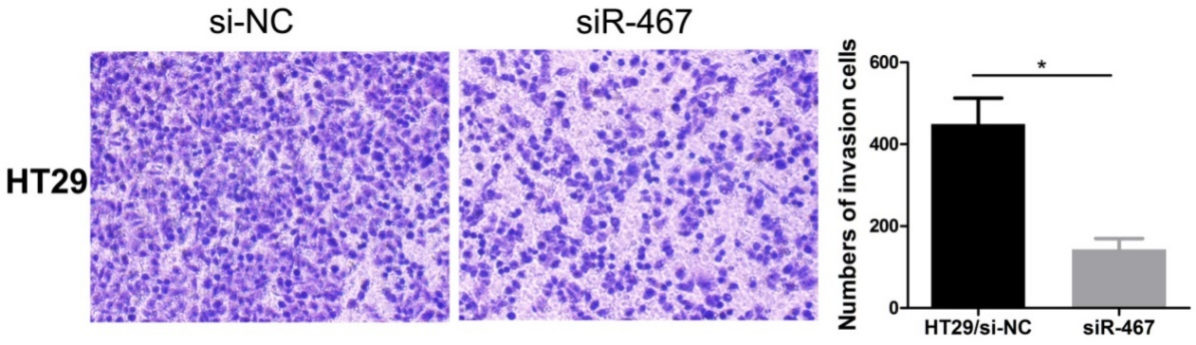
Knockdown of LINC00467 expression impeded the invasiveness of CRC cells. The invasive ability of HT29 cells was detected using a Transwell Matrigel assay after transfecting with si-NC or siR-467 for 48 h. Data are presented as mean ± SEM. *p < 0.05 compared with control.

Subsequently, we also explored the effect of *LINC00467* knockdown on the invasion of CRC cells. We detected the effect of *LINC00467* on the invasion of CRC cells using a Transwell Matrigel assay. The results indicated that knocking down *LINC00467* expression significantly inhibited the invasion capacity of HT29 cells compared to the control group (*p* < 0.05, Fig. 4).

### Knockdown of LINC00467 expression regulates proliferated and epithelial-mesenchymal transition (EMT) markers in CRC

**Figure 5:**
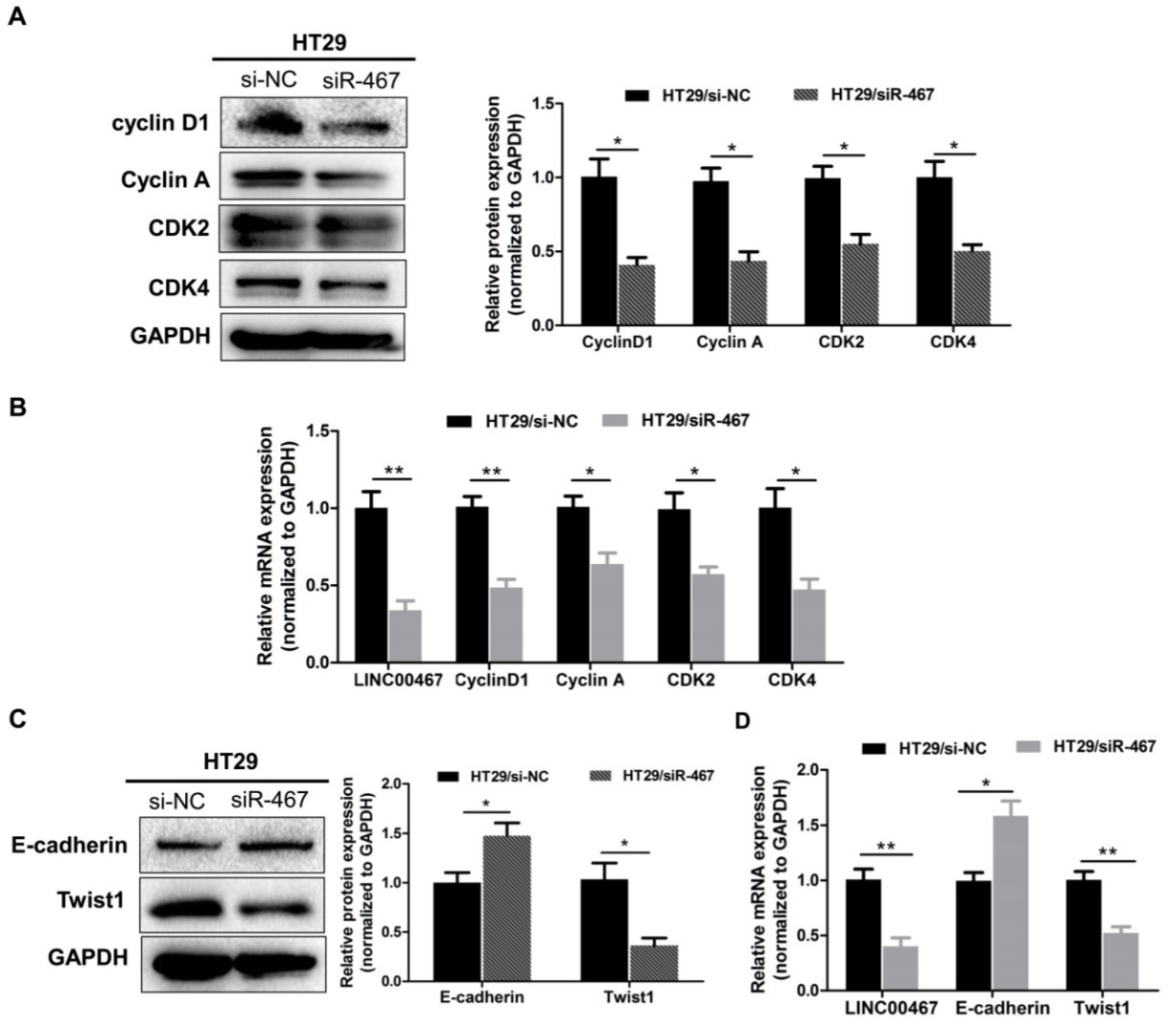
*LINC00467* knockdown inhibits the proliferation and EMT markers expressed in CRC. After transfecting with si-NC or siR-467 for 48 h, the effect of *LINC00467* on the protein and mRNA expressions of Cyclin D1, Cyclin A1, CDK2 and CDK4 (A-B) and of E‑cadherin and Twist1 (C-D) in HT29 cells were analysed by western blotting, densitometry and qRT-PCR. Data are presented as the mean ± SEM. *p < 0.05, **p < 0.01 compared with the control.

To further decipher the molecular mechanism by which knocking down *LINC00467* expression suppressed the proliferation and invasion of CRC cells *in vitro*, we assessed the mRNA and protein level of the proliferated markers Cyclin D1, Cyclin A1, CDK2 and CDK4, as well as, the epithelial marker E‑cadherin, and the mesenchymal markers Twist1 in HT29 cell lines, using qRT-PCR and western blotting techniques. Knocking down *LINC00467* significantly inhibited the expression of the proliferated markers Cyclin D1, Cyclin A1, CDK2 and CDK4 (*p* < 0.05, Fig. 5A-B). Meanwhile, *LINC00467* knockdown significantly reduced the expression of mesenchymal markers Twist1 and enhanced the expression of the epithelial marker E-cadherin (*p* < 0.05, Fig. 5C-D), thereby suppressing progression of epithelial-mesenchymal transition (EMT). Moreover, by analysing the GSE37364 datasets, we found that cyclin D1, CDK4, and Twist1 were significantly up-regulated in CRC tissues, whereas E-cadherin was significantly down-regulated in CRC tissues when compared with the non-tumour tissues (all *p* < 0.05, Supplemental Fig. 1). These results indicated that *LINC00467* may contribute to regulating the expression of proliferated and EMT marker expression in the CRC cells.

## Discussion

CRC is the third most prevalent cancer worldwide and it substantially affects human health 29. In 2015, 777,987 new cases and 352,589 deaths occurred due to CRC 30. With the development of basic and clinical investigations, the mechanisms of CRC and therapeutic strategies involved have become better understood 31. Nevertheless, due to postsurgical recurrence and metastasis of primary tumours, CRC mortality and morbidity rates remain high. Therefore, it is important to identify novel molecular therapeutic targets for CRC diagnosis, screening and therapy.

Recently, substantial evidence has demonstrated that lncRNAs are crucial in regulating various biological processes by modulating gene expression at the epigenetic, transcriptional and posttranscriptional levels 12, 32, thereby impacting the progression and development of several diseases, particularly cancer. Recent studies report that the disruption or disabling of lncRNAs levels closely correlates with the cancer cell proliferation and apoptosis, epithelial-mesenchymal transition (EMT) and drug resistance 33-36. Meanwhile, lncRNAs, including MALAT1 37, HOTAIR 38, GAS5 39, NEAT1 40, and TUG1 18, have been demonstrated to be differentially expressed and indicate poor prognosis. lncRNAs are considered as a kind of deal biomarker for tumour diagnosis and tumour recurrence surveys because they detect with high specificity and sensitivity, are easier to extract and exist steadily in blood and tissue 41.

With the popularity of gene microarray technologies and high-throughput sequencing, an increasing number of public databases (eg, TCGA, Oncomine, GEO, *etc*) have become powerful tools for predicting and identifying valuable lncRNAs 15. In this study, we analysed the lncRNAs that were found to be dysregulated in CRCs in previously published online GEO datasets (#GSE22598 and GSE37364), based on the Affymetrix Human Genome U133 Plus 2.0 platform, and found one novel lncRNA (*LINC00467*), which was significantly overexpressed in the two CRC datasets. *LINC00467* is located in the chr1q32.3 region and is 2616 nt long, and has only been previously reported to be found in the neuroblastoma 42. In our study, we aimed to explore the properties of *LINC00467* as a novel biomarker for CRC diagnosis and prognosis. We report here that *LINC00467* expression in CRC tissues was significantly higher than those in the matched adjacent normal tissues. Meanwhile, *LINC00467* overexpression in CRC patients had poorer RFS and OS rates, which may be an independent poor prognostic factor for the CRC patients, as suggested by multivariate analysis results.

Moreover, the biological functions and molecular mechanism of *LINC00467* have been seldom reported. Only one study by Atmadibrata 42 reported that in neuroblastoma, N-Myc targeting *LINC00467* could reduce the expression of tumour suppressor gene Dickkopf-related protein 1 (DKK1), thereby promoting neuroblastoma cell survival 42. However, the effect of *LINC00467* on the tumourigenesis of other tumours is poorly understood. In this study, our results demonstrate that knocking down *LINC00467* in HT29 cell lines could suppress the proliferation and invasiveness of CRC cells. Furthermore, we have shown that subcellular localization of *LINC00467* in the CRC cells was majorly located at the cytoplasm. Subcellular localization of lncRNAs is usually related to their biological function 43. Therefore, we speculated that the cytoplasmic expression of *LINC00467* in the CRC cells may sponge with miRNA to form the ceRNA, thereby regulating the malignant biological behaviour of CRC. In our next study, we will further explore the regulatory mechanism of *LINC00467* in CRC progression.

In conclusion, we found that a novel lncRNA,* LINC00467,* was the most significantly up-regulated lncRNA in CRC. Elevated *LINC00467* expression was associated with poor survival time (RFS and OS), and multivariate analysis results indicated that a high* LINC00467* expression level is an independent risk factor for CRC patients. We also demonstrated that knockdown of *LINC00467* could mediate the proliferation and invasion of CRC cells, thereby indicating that further investigation and study of *LINC00467* may lead to the development of novel CRC therapies.

## Supplementary Material

Supplementary figure S1.

## Figures and Tables

**Table 1:** Primer sequence for real-Time PCR

Gene	Primer (Forward)	Primer (Reverse)
*LINC00467*	ATTGAAGATGCTGCCAAGGG	GCCCAGTTTCAGTCCCTCTT
*CyclinD1*	TCGTTGCCCTCTGTGCCACA	GCAGTCCGGGTCACACTTGA
*CyclinA1*	GAGGTCCCGATGCTTGTCAG	GTTAGCAGCCCTAGCACTGTC
*CDK2*	AGCTGTGGACATCTGGAGCCTG	CCCAACCTTGTGATGCAGCCA
*CDK4*	TTGGTGTCGGTGCCTATGGG	CCATCAGCCGGACAACATTGG
*E-cadherin*	TGAAGCCCCCATCTTTGTGC	GGCTGTGTACGTGCTGTTCT
*Twist1*	CAGCGCACCCAGTCGCTGAA	CCAGGCCCCCTCCATCCTCC
*18S*	GTGGGCCGAAGATATGCTCA	TTGGCTAGGACCTGGCTGTA
*U6*	CTCGCTTCGGCAGCACA	AACGCTTCACGAATTTGCGT
*GAPDH*	AACGGATTTGGTCGTATTGG	TTGATTTTGGAGGGATCTCG

**Table 2:** The expression of *LINC00467* in TCGA Colon adenocarcinoma (COAD) RNA-seq dataset were analyzed by the Cancer RNASeq Nexus.

	*LINC00467* (Transcript ID: uc001hil.3)		
Colon adenocarcinoma subset pair	Average expression in cancer	Average expression in normal	Cancer versus Normal *P*-value
Colon adenocarcinoma--Stage I versus Normal (adjacent normal)	8.00	5.06	*P* <0.05
Colon adenocarcinoma--Stage II versus Normal (adjacent normal)	6.75	5.06	
Colon adenocarcinoma--Stage IIA versus Normal (adjacent normal)	8.18	5.06	
Colon adenocarcinoma--Stage IIB versus Normal (adjacent normal)	11.83	5.06	
Colon adenocarcinoma--Stage III versus Normal (adjacent normal)	6.15	5.06	
Colon adenocarcinoma--Stage IIIA versus Normal (adjacent normal)	7.22	5.06	
Colon adenocarcinoma--Stage IIIB versus Normal (adjacent normal)	8.88	5.06	
Colon adenocarcinoma--Stage IIIC versus Normal (adjacent normal)	8.43	5.06	
Colon adenocarcinoma--Stage IV versus Normal (adjacent normal)	7.95	5.06	

**Note:** The Cancer RNASeq Nexus (CRN, http://syslab4.nchu.edu.tw/CRN) is an open resource for intuitive data exploration, providing coding-transcript/lncRNA expression profiles that contain alternative splicing to support researchers generating new hypotheses in cancer research and personalized medicine.
