# Supplementary material for: Up-regulation of LINC00467 promotes the tumourigenesis in colorectal cancer
Source: J Cancer. 2019 Oct 19;10(25):6405–13. doi: 10.7150/jca.32216 (PMC6856745; doi:10.7150/jca.32216)
Supplement: Supplementary file 1 — Supplementary figure S1. [file jcav10p6405s1.pdf]

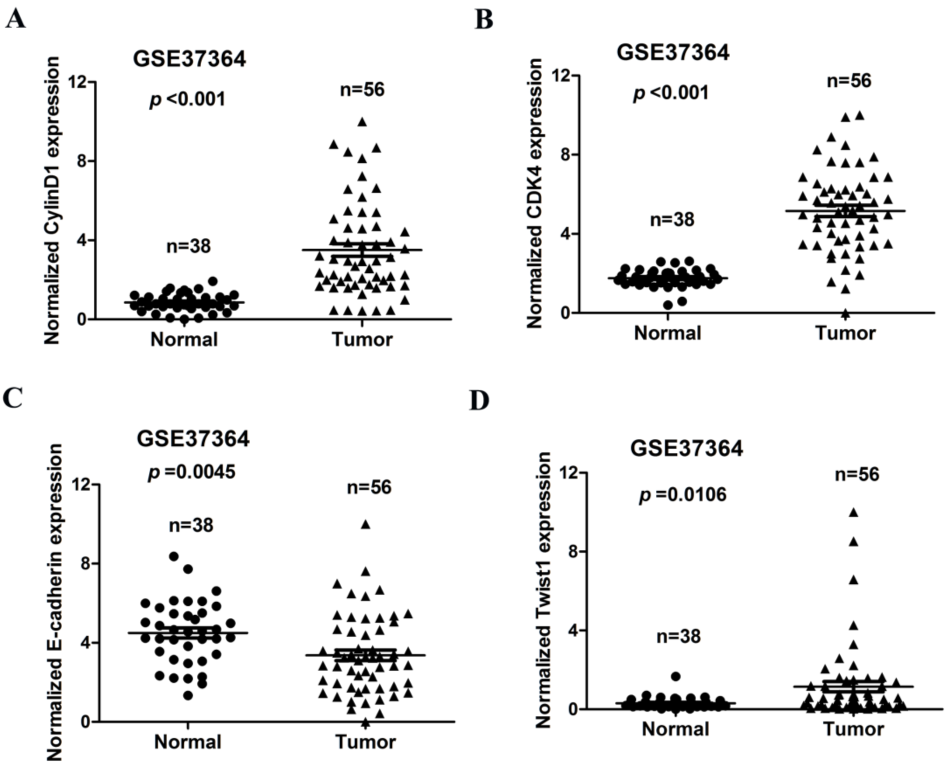

Supplemental figure 1. #GSE37364 (38 normal colon samples and 56 primary CRC samples) from GEO database were used to analyse the expression of *CyclinD1*(A), *CDK4*(B), *E-cadherin*(C), *Twist1*(D). Data are presented as mean ± SEM $*p < 0.05$, $**p < 0.01$, $***p < 0.001$ compared with control.
